# Site-specific ADP-ribosylation of histone H2B in response to DNA double strand breaks

**DOI:** 10.1038/srep43750

**Published:** 2017-03-02

**Authors:** Alina Rakhimova, Seiji Ura, Duen-Wei Hsu, Hong-Yu Wang, Catherine J. Pears, Nicholas D. Lakin

**Affiliations:** 1 Department of Biochemistry, University of Oxford, South Parks Road, Oxford OX1 3QU, UK

## Abstract

ADP-ribosyltransferases (ARTs) modify proteins with single units or polymers of ADP-ribose to regulate DNA repair. However, the substrates for these enzymes are ill-defined. For example, although histones are modified by ARTs, the sites on these proteins ADP-ribosylated following DNA damage and the ARTs that catalyse these events are unknown. This, in part, is due to the lack of a eukaryotic model that contains ARTs, in addition to histone genes that can be manipulated to assess ADP-ribosylation events *in vivo*. Here we exploit the model *Dictyostelium* to identify site-specific histone ADP-ribosylation events *in vivo* and define the ARTs that mediate these modifications. *Dictyostelium* histones are modified in response to DNA double strand breaks (DSBs) *in vivo* by the ARTs Adprt1a and Adprt2. Adprt1a is a mono-ART that modifies H2BE18 *in vitro*, although disruption of this site allows ADP-ribosylation at H2BE19. Although redundancy between H2BE18 and H2BE19 ADP-ribosylation is also apparent following DSBs *in vivo*, by generating a strain with mutations at E18/E19 in the *h2b* locus we demonstrate these are the principal sites modified by Adprt1a/Adprt2. This identifies DNA damage induced histone mono-ADP-ribosylation sites by specific ARTs *in vivo*, providing a unique platform to assess how histone ADP-ribosylation regulates DNA repair.

ADP-ribosyltransferases (ARTs) are primary sensors of DNA damage that catalyse the addition of ADP-ribose onto target proteins. 17 genes containing predicted ART catalytic domains have been identified in humans^1^ and the majority of these add single ADP-ribose moieties onto target proteins by a process known as mono-ADP-ribosylation (MARylation). However, several ARTs, including PARP1, PARP2, PARP5a and PARP5b catalyse ADP-ribose polymers that contain both linear and branched glycosidic linkages^2^,^3^. Whilst ADP-ribosylation has been implicated in a number of cellular processes including cell growth and differentiation, transcriptional regulation and programmed cell death^3^, its best defined role is in regulating DNA repair, with specific reference to DNA strand breaks. PARP1 and PARP2 are primary sensors of DNA damage that become activated on binding to DNA single strand breaks (SSBs) and modify a variety of substrates, including themselves, to facilitate accumulation of SSB repair factors at the break site^4^,^5^,^6^,^7^,^8^,^9^,^10^,^11^,^12^. ARTs have also been implicated in regulating DNA double strand break (DSB) repair by homologous recombination (HR) and non-homologous end-joining (NHEJ). PARP1 is required for HR-mediated restart of damaged and/or stalled replication forks^13^,^14^,^15^. It also promotes alternative-NHEJ (alt-NHEJ), an end-joining pathway activated in the absence of core NHEJ factors^16^,^17^,^18^,^19^. Whilst PARP1 has been implicated in regulating core NHEJ^20^,^21^, PARP3 promotes this repair pathway by facilitating accumulation of APLF and Ku at damage sites^22^,^23^,^24^,^25^,^26^.

Whilst the role of ARTs in DNA repair is well established, the identity of proteins ADP-ribosylated in response to DNA damage is less clear. Recent advances in mass spectrometry have begun to define the ADP-ribosylome^27^,^28^,^29^,^30^,^31^,^32^. However, the specific sites on these proteins modified *in vivo* and the ARTs that catalyse these events are largely unknown. This situation is exemplified by histones^33^. A fraction of all core histones isolated from cell nuclei are ADP-ribosylated^34^,^35^,^36^,^37^,^38^ and H2B/H3 are the principal histones modified in response DNA alkylating agents^39^,^40^. However, whether a similar pattern of histone ADP-ribosylation occurs in response to other varieties of DNA damage is unclear. Moreover, although the sites on histones modified by PARP1 have been established *in vitro*^41^, which sites on histones are ADP-ribosylated in response to DNA damage *in vivo* and the ARTs that catalyse these events are unknown. This, in part, is due to the lack of an appropriate eukaryotic experimental platform in which ARTs and histone genes can be manipulated to characterise ADP-ribosylation sites identified by biochemical or MS analysis. For example, high copy number arrays of histone genes in vertebrates makes it difficult to genetically manipulate these loci to assess how specific histone ADP-ribosylation events regulate a variety of cellular processes. This issue is compounded by the absence of ARTs in invertebrate models where histones genes are amenable to genetic manipulation, precluding an analysis of these pathways in these experimental systems.

Given these considerations, there is a call for a genetically tractable eukaryotic model that exhibits conservation of ARTs and key vertebrate DNA repair pathway components, in addition to single copy histone genes amenable to gene disruption and replacement strategies. All of these criteria are uniquely met in the eukaryotic amoeba *Dictyostelium*. Importantly, this organism has diverged less from humans than other invertebrates^42^. This is particularly striking with regards to DNA repair and several key human DNA repair pathway components are conserved in this organism that are absent in other models commonly used to study these pathways^43^,^44^,^45^,^46^,^47^,^48^,^49^,^50^,^51^. Importantly, the *Dictyostelium* genome contains 15 proteins with predicted ART catalytic domains^47^ and similar to vertebrates, two (Adprt1b and Adprt2) confer tolerance of cells to SSBs^43^,^52^. Moreover, in parallel with studies in mammalian cells^22^,^24^,^26^, we identified a further ART (Adprt1a) that whilst dispensable for SSBR, is required to promote NHEJ by facilitating accumulation of Ku at DSBs through a PAR interaction domain (PID) located in Ku70^43^,^47^.

*Dictyostelium* is ideally suited to assess how post-translational modification of histone proteins regulates a variety of cellular processes. Although, *Dictyostelium* possess the standard core histones, they exhibit a greater diversity of histone variants than budding yeast whilst retaining a simpler complement than metazoa, facilitating their analysis by both biochemical and genetic approaches^53^,^54^,^55^. The major post-translational modifications on histone N-terminal tails, including phosphorylation, acetylation and methylation, in addition the enzymes that catalyse these reactions, are also conserved in *Dictyostelium*^53^,^55^,^56^,^57^,^58^. Critically, in contrast to vertebrates, *Dictyostelium* contains single copies of most histone genes^53^,^55^, making their genetic manipulation relatively straightforward. Here we exploit the ability to manipulate histone genes in *Dictyostelium*, in addition to the conservation of ARTs in this organism, to assess DNA damage-induced site-specific ADP-ribosylation events on histones *in vivo* and define the ARTs that mediate these modifications. These data demonstrate, for the first time, mapping and confirmation of DNA damage induced histone ADP-ribosylation sites by a specific ARTs *in vivo* and will provide a unique experimental platform to assess the role of histone ADP-ribosylation in DNA repair and other cellular process.

## Results

### ADP-ribosylation of *Dictyostelium* histone proteins in response to DNA DSBs

**Figure 1:**
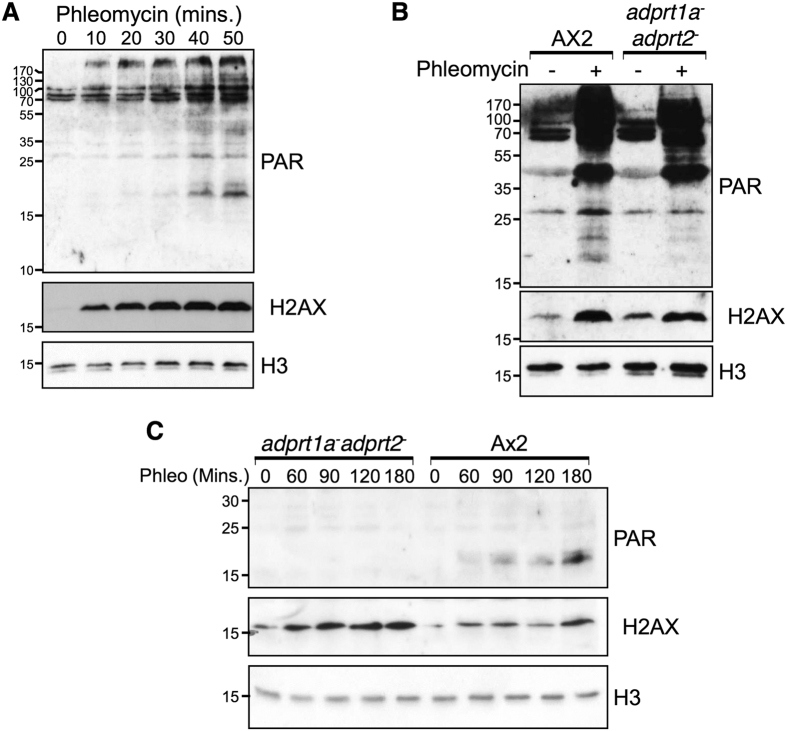
ADP-ribosylation of chromatin associated proteins in response to DSBs is dependent on Adprt1a and Adprt2. (**A)** Ax2 cells were left untreated or exposed to 300 μg/ml phleomycin for the indicated times. Chromatin fractions were prepared and western blotting performed using the indicated antibodies. (**B**) Ax2 or *adprt1a*^*−*^*adprt2*^*−*^ cells were left untreated or exposed to 300 μg/ml phleomycin for 60 minutes as indicated. Chromatin fractions were prepared and western blotting performed using the indicated antibodies. (**C**) Ax2 cells or the *adprt1a*^*−*^*adprt2*^*−*^ strain were exposed to phleomycin for the times indicated. Histone-enriched acid extracts were prepared and western blotting performed with antibodies as indicated.

Previously, we identified that nuclear ADP-ribosylation is required for effective NHEJ in *Dictyostelium* and that this is dependent on the ARTs Adprt1a and Adprt2^43^. To address which chromatin-associated proteins are targeted by these enzymes following DSBs, chromatin fractions were prepared from *Dictyostelium* Ax2 cells following DSB induction by phleomycin and ADP-ribosylation assessed by western blotting using an antibody that recognises PARylated proteins. PARylation of chromatin-associated proteins is induced in a time-dependent manner in Ax2 cells exposed to phleomycin (Fig. 1A) and several of these proteins migrate with molecular weights similar to histones (15–25 kDa). Our previous work illustrated that nuclear ADP-ribosylation is dependent on both Adprt1a and Adprt2^43^. Therefore, we considered whether these ARTs are required for DSB-induced ADP-ribosylation of chromatin-associated proteins within the 15–25 kDa range. Similar to nuclear ADP-ribosylation^43^, disruption of Adprt1a or Adprt2 alone reduced ADPr of these proteins (data not shown) whilst these events were almost completely lost in the *adprt1a*^*−*^*adprt2*^*−*^ strain (Fig. 1B).

Next, we considered whether the PARylated proteins of between 15–25 kDa present in chromatin fractions following DSB-induction represent histone proteins. Consistent with this hypothesis, several PARylated polypeptides with molecular weights of between 15–25 kDa co-purify with histones during an optimised acid-extraction protocol developed to enrich *Dictyostelium* histones from vegetative cells (Fig. 1C)^55^. Similar to chromatin fractions, these proteins are absent in acid extracts prepared from *adprt1a*^*−*^*adprt2*^*−*^ cells, indicating these modifications are dependent on Adprt1a and Adprt2. Taken together, these data indicate that several chromatin-associated proteins are PARylated in response to DSBs in an Adprt1a/Adprt2-dependent manner and that a subset of these proteins likely represents histones.

### *Dictyostelium* H2B is ADP-ribosylated in response to DNA DSBs *in vivo*

**Figure 2:**
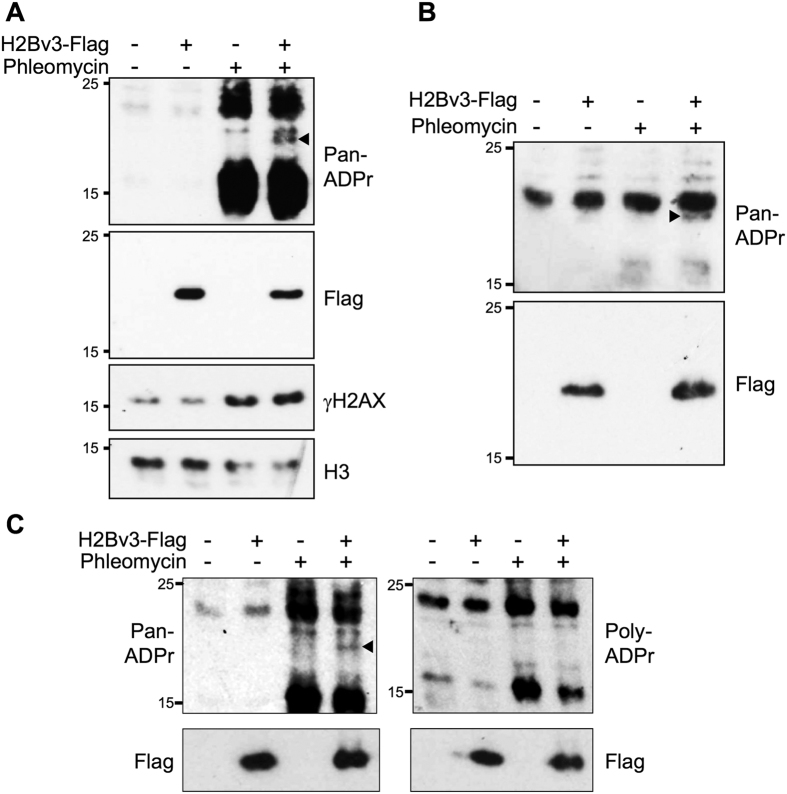
ADP-ribosylation of histone H2Bv3 in response to DNA DSBs. (**A**) Ax2 cells expressing H2Bv3-Flag, or control cells containing empty vector were left untreated or exposed to 300 μg/ml of phleomycin for 1 hour. Histone-enriched acid extracts were prepared and western blotting performed with the indicated antibodies. ADP-ribosylated H2Bv3-Flag is indicated with an arrow. (**B**) Ax2 cells expressing H2Bv3-Flag, or control cells containing empty vector, were treated as in (**A**). Proteins were released from chromatin and immunoprecipitated with Flag antibodies. Western blotting was performed with antibodies as indicated. ADP-ribosylated H2Bv3-Flag is indicated with an arrow. (**C**) Ax2 cells expressing H2Bv3-Flag, or control cells containing empty vector were treated as in (**A**). Western blotting was performed using a reagent that recognises both poly- and mono-ADP-ribosylation (left), or a reagent that recognises only poly-ADP-ribosylated proteins (right). ADP-ribosylated H2Bv3-Flag is indicated with an arrow.

Similar to *Dictyostelium* Adprt1a, human PARP3 promotes DSB repair by facilitating accumulation of NHEJ factors at damage sites^22^,^23^,^24^,^25^,^26^. All core histones are modified by PARP3 *in vitro*, although in the context of reconstituted nucleosomes H2B is the major histone modified by this ART^59^. Whether PARP3 is responsible for DNA damage-induced H2B ADP-ribosylation *in vivo* is unknown. Given Adprt1a is the functional equivalent of PARP3^43^,^52^, we considered whether H2B is ADP-ribosylated in response to DNA DSBs *in vivo*. H2Bv3 is the principal H2B variant expressed in vegetative *Dictyostelium*^53^,^55^. To test whether this histone is ADP-ribosylated in response to DNA DSBs *in vivo*, we expressed H2Bv3 with 3x tandem C-terminal Flag tags in Ax2 cells and monitored its ADP-ribosylation status following DNA DSBs. Inclusion of the epitope tags on H2Bv3 increases the molecular weight of the protein to distinguish it from endogenous H2Bv3 and other modified histones during SDS-PAGE analysis. Similar to previous observations using a PAR-specific antibody (Fig. 1C), several polypeptides of between 15–25 kDa are detected by a reagent that recognises both MARylated and PARylated proteins specifically in extracts prepared from cells following exposure to phleomycin (Fig. 2A). The majority of these modifications are independent of H2Bv3-Flag expression, most likely reflecting ADP-ribosylation of endogenous histone proteins (Fig. 2A). Strikingly, a further DSB-induced ADP-ribosylated protein is present only in cells expressing H2Bv3-Flag. Moreover, a protein of the predicted molecular weight of H2Bv3-Flag is detected specifically in Flag-immunoprecipitates from extracts prepared after exposure of H2Bv3-Flag expressing cells to phleomycin (Fig. 2B), confirming ADP-ribosylation of H2Bv3-Flag in response to DSBs. Whilst DSB-induced ADP-ribosylation of H2BV3-Flag is detectable using the reagent that recognises both MARylation and PARylation events, it is not recognised by a reagent that recognises only PARylated proteins (Fig. 2C). From these data we conclude that H2Bv3 is ADP-ribosylated in response to DNA DSBs *in vivo* primarily by MARylation.

### Adprt1a is a mono-ART that modifies the N-terminus of H2Bv3 *in vitro*

**Figure 3:**
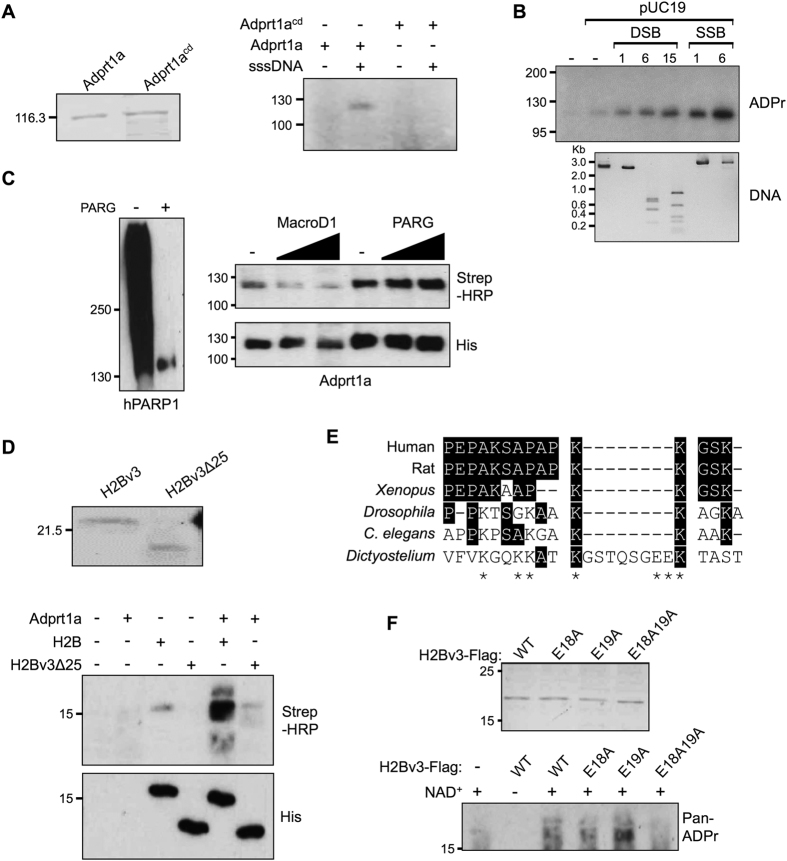
Adprt1a is a DNA strand break-induced mono-ART that ADP-ribosylates H2Bv3 at E18 and E19. (**A**) Wild-type or catalytic dead (Adprt1a^cd^) His-tagged Adprt1a was expressed and purified from bacteria (left) and employed in ADP-ribosylation assays using ^32^P-labeled NAD^+^ in the absence or presence of sheared salmon sperm DNA (sssDNA). Following SDS-PAGE, ADP-ribosylated proteins were detected by auto-radiography. (**B)** ADP-ribosylation assays were performed using His-Adprt1a as in (**A**) with the exception that pUC19 DNA either uncut, or cut with enzymes to produce the indicated numbers of breaks, was employed in assays instead of sssDNA. (**C)** His-Adprt1a (right) and human PARP1 (left) were auto-ribosylated by incubation with biotin-NAD^+^ prior to incubation with increasing concentrations of MacroD1 or PARG as indicated. ADP-ribosylated proteins were detected by Western blotting with Streptavidin conjugated HRP. (**D)** His-H2Bv3 or His-H2Bv3 lacking the N-terminal 25 amino acids (H2Bv3Δ25) were expressed and purified from bacteria (upper panel). ADP-ribosylation reactions were performed with Adprt1a in the presence of sssDNA as indicated. ADP-ribosylated proteins were detected as in (**C**). (**E)** Sequence alignment of the N-terminal 24 amino acids of *Dictyostelium* H2Bv3 with the equivalent regions of H2B from a variety of species. Identical amino acids are highlighted and potential ADP-ribosylation sites in the *Dictyostelium* protein indicated (*). (**F)** Wild-type His-H2Bv3, or His-H2Bv3 mutated at E18, E19, or both E18 and E19 were expressed and purified from bacteria (upper panel). Proteins were employed in ADP-ribosylation reactions as in (**D**).

Although advances in mass spectrometry have begun to identify ADP-ribosylated proteins^27^,^28^,^29^,^30^,^31^,^32^, mapping site specific ADP-ribosylation events *in vivo* remains challenging. Given Adprt1a is the principle ART required for DSB-induced ADP-ribosylation^43^, we first considered the catalytic properties of Adprt1a *in vitro* and whether it is capable of modifying H2Bv3. It is well established that human DNA damage responsive ARTs are capable of auto-ADP-ribosylation *in vitro* and that this activity is activated by a variety of DNA strand breaks^60^. Similarly, we observe an ADP-ribosylated species that co-migrates with recombinant Adprt1a but not a catalytic-dead version of the protein (Adprt1a^cd^) only in reactions that contain sheared salmon sperm DNA (Fig. 3A), indicating Adprt1a is activated by DNA to undergo auto-ADP-ribosylation *in vitro*. Given sheared DNA contains different DNA structures, most notably a variety of DNA single and double strand breaks, we next considered whether Adprt1a is activated by a specific variety of DNA strand break. Similar to human PARP3^61^,^62^, Adprt1a is effectively activated by DNA DSBs and SSBs and enzyme activity increases in proportion to the number of breaks present in reactions (Fig. 3B). We also established whether Adprt1a auto-ribosylation represents MARylation or PARylation events. PARG removes Poly-ADP-ribose polymers from proteins^63^,^64^, whilst MacroD1 removes mono-ADP-ribose^65^,^66^,^67^. Incubation of auto-ADP-ribosylated Adprt1a with PARG does not significantly impact of the levels of auto-ribosylated Adprt1a, whilst inclusion of MacroD1 reduces levels of ADP-ribosylated Adprt1a (Fig. 3C). From these data we concluded that Adprt1a is a mono-ART that is activated by DNA strand breaks *in vitro*.

Next, we established whether H2Bv3 is modified by Adprt1a *in vitro* and at which sites. Given human PARP1 is able to modify the N-terminus of all core histones *in vitro*^41^, we first tested whether full length H2Bv3, or H2Bv3 lacking the N-terminal 25 amino acids of the protein, are modified by Adprt1a (Fig. 3D). Full-length H2Bv3 is effectively ADP-ribosylated by Adprt1a *in vitro* and this is dependent on DNA and Adprt1a catalytic activity (Appendix Figure S1). Deletion of the N-terminal 25 amino acids of H2Bv3 almost completely ablates this modification (Fig. 3D), indicating the major ADP-ribose acceptor sites reside in this region of the protein. Although ARTs modify proteins at aspartate, glutamate, arginine, lysine, cysteine and asparagine amino acids^1^, the N-terminal 25 amino acids of *Dictyostelium* H2Bv3 contain only 5xLys and 2xGlu amino acids that are potential ADP-ribose acceptor sites (Fig. 3E). Given MacroD1 removes ADP-ribose moieties from glutamates^65^,^66^,^67^, and that this protein reverses Adprt1a catalysed ADP-ribosylation events (Fig. 3C), we hypothesised that E18 and/or E19 are modified by this enzyme. Therefore, we expressed and purified H2Bv3 from bacteria in which E18 and E19 have been mutated to Ala either alone or in combination, and assessed their ability to undergo Adprt1a-mediated ADP-ribosylation *in vitro*. Mutation of E18 of H2Bv3 has only a slight impact on Adprt1a-mediated ADP-ribosylation, whilst mutation of E19 increases the ability of Adprt1a to modify H2Bv3 (Fig. 3F). However, mutation of both E18 and E19 in combination dramatically reduces Adprt1a-mediated ADP-ribosylation of H2Bv3. These data demonstrate that whilst E18 of H2Bv3 is ADP-ribosylated by Adprt1a *in vitro*, when this site is lost E19 is instead modified.

### E18 and E19 of H2Bv3 are ADP-ribosylated in response to DNA DSBs *in vivo*

**Figure 4:**
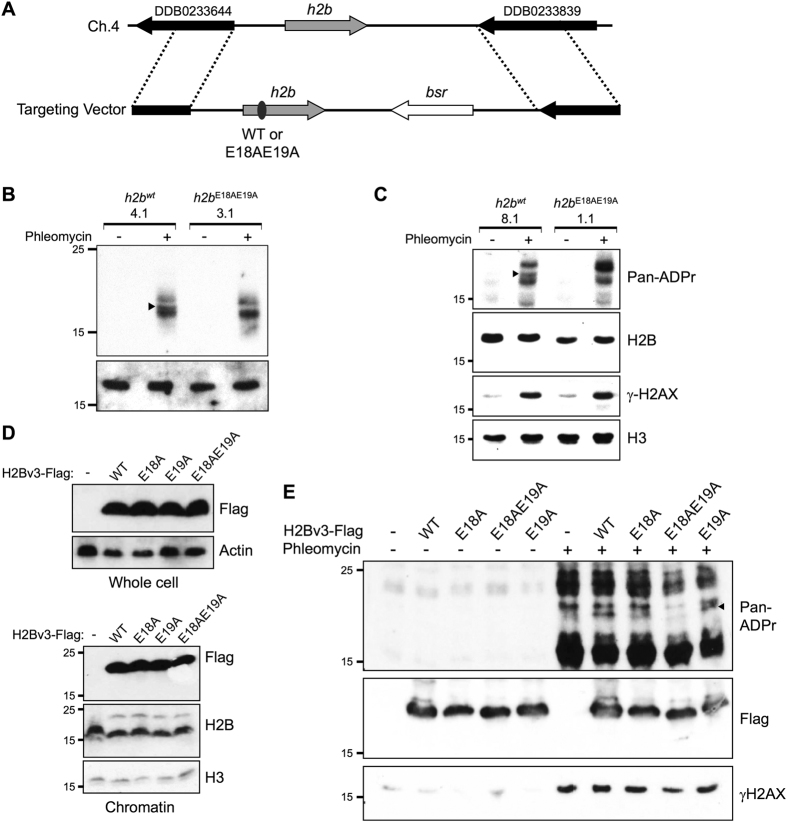
H2Bv3 is ADP-ribosylated at E18 and E19 *in vivo*. (**A**) Gene replacement strategy to replace the endogenous *h2b* locus with either wild-type *h2b (h2b*^*wt*^), or *h2b* containing E18AE19A mutations (*h2b*^*E18AE19A*^). (**B** and **C**) Two independent *h2b*^*wt*^ and *h2b*^*E18AE19A*^ strains were left untreated or exposed to 300 μg/ml phleomycin for 60 minutes as indicated. Histone-enriched acid extracts were prepared and western blotting performed using the indicated antibodies. Arrow heads indicate the position of ADP-ribosylated H2B protein in *h2b*^*wt*^ cells. (**D)** Wild-type H2Bv3-Flag or H2Bv3-Flag with the indicated mutations were expressed in Ax2 cells. Whole cell or chromatin extracts were prepared and western blotting performed with the indicated antibodies. (**E)** Ax2 cells expressing wild-type H2Bv3-Flag or the indicated H2Bv3-Flag mutants were left untreated or exposed to 300 μg/ml of phleomycin for 1 hour as indicated. Histone-enriched acid extracts were prepared and western blotting performed with the indicated antibodies. ADP-ribosylated H2Bv3-Flag is indicated with an arrow.

We next assessed whether the ability of Adprt1a to modify H2Bv3 at E18 and E19 *in vitro* is reflected in these amino acids being targets for DSB-induced ADP-ribosylation *in vivo*. In contrast to vertebrates, *Dictyostelium* contain single copies of all histone genes, with the exception of H4, of which there are two copies^53^,^55^. Therefore, we exploited gene replacement technology to manipulate the endogenous *h2bv3* locus at the ADP-ribosylation sites identified *in vitro* (Fig. 4A and Appendix Figure S2) and assessed the impact of these mutations on DSB-induced H2Bv3 ADP-ribosylation. Accordingly, a gene replacement vector was designed containing the *h2b* gene that whilst under the control of its own promoter, contains E18A and E19A mutations (*h2bv3*^*E18AE19A*^). Given this strategy results in strains containing some residual vector sequence, as a control we generated an equivalent strain, but with a wild-type version of the *h2Bv3* gene replacing the endogenous locus (*h2bv3*^*wt*^).

The resulting *h2bv3*^*wt*^ and *h2bv3*^*E18AE19A*^ strains were exposed to phleomycin and ADP-ribosylation assessed using a reagent that detects both MARylation and PARylation. Using high resolution SDS-PAGE, three protein species of between 15–25 kDa are robustly ADP-ribosylated in response to DSBs in two independent *h2bv3*^*wt*^ strains (Fig. 4B and C). The intensity of the middle of these three bands, which migrates with a similar molecular weight to H2Bv3, is dramatically reduced in two independent *h2bv3*^*E18AE19A*^ strains, indicating loss of H2Bv3 ADP-ribosylation. Whilst these data clearly reveal that H2Bv3 is ADP-ribosylated at E18/E19 *in vivo*, they are unable to distinguish whether there is a preference for either E18 or E19 to be ADP-ribosylated in response to DSBs. To address this question we generated Ax2 strains that express H2Bv3-Flag containing E18A, E19A or E18AE19A mutations and assessed the ability of these proteins to be ADP-ribosylated in response to DSBs *in vivo*. All H2Bv3-Flag variants are expressed at similar levels and effectively incorporated into chromatin (Fig. 4D). Consistent with previous observations (Fig. 2), we observe robust DNA damage-induced ADP-ribosylation of a band in H2Bv3-Flag expressing cells that is absent from cells that do not express the protein, confirming the identity of this band as H2Bv3-Flag (Fig. 4E). Whilst the E19A mutation has little impact on ADP-ribosylation status of this protein, the E18A mutation slightly reduces its modification. In contrast, mutation of both E18 and E19 dramatically reduces ADP-ribosylation of H2Bv3-Flag (Fig. 4E). Taken together, these data indicate that similar to our observations *in vitro*, E18 is the preferred site on H2Bv3 ADP-ribosylated in response to DNA DSBs *in vivo*, although in its absence E19 is also able to be modified. Moreover, the almost complete loss of H2Bv3 ADP-ribosylation following mutation of E18 and E19 indicate no other sites on the protein are robustly ADP-ribosylated following DSBs.

## Discussion

*Dictyostelium* Adprt1a mediates DSB-induced ADP-ribosylation to promote NHEJ^43^. Similarly, human PARP3 has also been implicated in regulating NHEJ by facilitating accumulation of APLF and Ku at damage sites^24^,^26^. However, the substrates modified by Adprt1a or PARP3 *in vivo* are unknown. The substrates targeted by other ARTs are similarly unclear. For example, recent advances in mass spectrometry have identified a number of proteins, including histones that are ADP-ribosylated in response to genotoxic agents^31^,^32^. Although modification of these substrates is disrupted by PARP inhibitors, these compounds target multiple ARTs^68^, making it difficult to attribute these events to a specific ART(s). Our data indicate histones are likely targets for ADP-ribosylation in response to DNA DSBs and we demonstrate that H2B is modified at E18 and E19 in response to this variety of DNA damage. We observe co-fractionation of several ADP-ribosylated proteins with histones during an optimised acid extraction procedure, and through manipulation of the endogenous *h2b* locus, identify that one of these modifications represents ADP-ribosylation of H2B at E18/E19. Given ADP-ribosylation of acid extracted histones is dependent on Adprt1a and Adprt2; we concluded that modification of H2B at E18/E19 in response to DSBs is dependent on these ARTs.

Histones are known targets for ADP-ribosylation. All core histones, in addition to H1, are modified in response to DNA alkylating agents^39^,^40^,^69^. However, whether similar patterns of histone ADP-ribosylation are observed in response to other DNA damage types and the sites modified is unclear. We only observe robust ADP-ribosylation of H2B at E18/E19 following exposure of cells to DNA DSBs, suggesting little modification of this site in the absence of genotoxic stress. Isolation of H2B from rat nuclei following incubation with radio-labelled NAD + identified E2 of H2B as an ADP-ribosylation site, although these experiments were performed in the absence of DNA damaging agents^37^. It is tempting to speculate that whilst E18 and E19 of *Dictyostelium* H2B are not absolutely conserved in vertebrates (Fig. 3E), E2 of human H2B might be analogous to these sites. Given the susceptibility of inducing DNA damage and ADP-ribosylation during cell extraction procedures^31^, it is possible the ADP-ribosylation of rat H2B observed previously is a reflection of DNA damage induced during sample preparation, or low levels of endogenous DNA damage present in cells in the absence of genotoxins. In further support of E2 of human H2B being equivalent to the ADP-ribosylation sites identified here, PARP3 specifically modifies H2B at this site in reconstituted nucleosomes *in vitro*^62^. Together, these observation may indicate that ADP-ribosylation sites do not have to be absolutely conserved to be functionally important, but rather reside in equivalent regions of the protein. Interestingly, we observe that disruption of E19 increases ADP-ribosylation of H2B by Adprt1a *in vitro*, presumably at E18 given the E18AE19A mutant is not significantly modified in these assays (Fig. 3F). Whilst it is interesting to speculate that E19 may quench ADP-ribosylation of H2B at E18, this observation is not apparent when assessing the ADP-ribosylation status of the equivalent mutants *in vivo* (Fig. 4E). Nevertheless, both *in vitro* and *in vivo* assays reveal that whilst mutation of E18 reduces ADP-ribosylation of H2B by Adprt1a, a significant reduction in its modification status is only observed when E18 and E19 are mutated in combination (Figs 3F and 4E). These observations underscore redundancy and further plasticity in ADP-ribosylation events that has implications regarding the design of protein mutations to study these modifications.

In further support of Adprt1a directly modifying H2B at E18/E19, we provide evidence that H2B is MARylated at E18/E19 in response to DNA DSBs. It is interesting to note, however, that a number of polypeptides in histones preparations are ADP-ribosylated following DNA DSBs in the *h2b*^*E18AE19A*^ strains, suggesting that similar to other organisms several *Dictyostelium* histones other than H2B are ADP-ribosylated in response to DNA damage. ADP-ribosylation promotes the enrichment of chromatin remodelling and repair factors at DNA lesions through ADP-ribose interaction domains^70^. Additionally, different ADP-ribose interaction domains recognise different modification types. For example, whilst the PBZ domain recognises PAR chains, the macro domain is able to recognise MARylated proteins^70^,^71^. To date, it is unknown whether DNA damage induces MARylation or PARylation of histones, or a combination of the two at different sites to influence differential recruitment of repair factors to DNA lesions. In this regard, although Adprt1a and the PBZ domain of Ku70 are required for accumulation of Ku at DSBs and efficient NHEJ^43^, we do not observe sensitivity of the *h2b*^*E18AE19A*^ mutants to DSBs during spore germination (Appendix Figure S3), a stage of the *Dictyostelium* life cycle where cells become reliant on this repair pathway^46^. Therefore, it will be important to establish whether ADP-ribosylation of different histone sites, or with a specific type of ADP-ribosylation event, is a determining factor in influencing which factors are recruited to a particular type of DNA lesion. The availability of *Dictyostelium* as a model in which histone ADP-ribosylation sites can be manipulated will help resolve these questions.

## Materials and Methods

### Cell culture and strain generation

*Dictyostelium* cells were grown according to standard procedures, either axenically or on SM agar plates in association with *Klebsiella aerogenes*. The *adprt1a*^*−*^, *adprt2*^*−*^ and *adprt1a*^*−*^*adprt2*^*−*^ strains have been previously described^43^.

To construct the targeting vector for *h2bv3* gene replacement, *h2bv3* coding sequence, in addition to regions flanking the gene (Chromosome 4; position 453224 to 456713), was amplified by PCR from AX2 genomic DNA using the primers 5′-CTTATACGATCGACTGATGCTGTAACAATAG-3′ and 5′-CCATATCATGGTGGATATTACCATGGTC-3′. The amplified fragment was first subcloned into pJET 1.2 (Thermo Scientific). The selection marker (BSR cassette) was excised from pLPBLP^72^ using SmaI, and inserted to a BsrGI site in the 3′ non-coding region downstream of *h2bv3* gene. To eliminate unnecessary restriction sites of pJET 1.2 vector, the whole *h2bv3* gene and flanking regions, including the selection marker, was cut using BspHI, treated by Klenow fragment to fill in ends, and then cut with NotI. This fragment was then inserted into pBluescriptII SK- (Agilent Technologies) at HindIII (treated by Klenow fragment), and NotI sites.

To introduce E18 and E19 to A mutations into the targeting vector, a short fragment containing 5′ non-coding region, and 5′ sequences of *h2bv3* coding region was amplified by PCR using 5′-CAATAAAsATTACACTCCTAA-3′ and 5′-CAGAAGCTTCGGTGGCGATTCTGT-3′ primers, and then subcloned into pJET 1.2 vector. E18 and E19 to A mutation was introduced by QuickChange method (Agilent Technologies) using a 5′-CAAAGGTTCAACTCAATCCGGAGCAGCGAAAACCGCTTCAACCAC-3′ primer, and corresponding anti-sense primer. The mutagenesis primer also carried a BspEI restriction enzyme site introduced into the *h2bv3* gene without changing its amino acid sequence to facilitate screening of mutants. The short AfeI and HindIII fragment carrying the mutation was used to replace the corresponding sequence in the targeting vector.

Both targeting vectors carrying wildtype and mutated *h2bv3*, were then transfected to AX2 cells by electroporation and selected under 10 μg/ml blasticidin for two weeks. Blasticidin resistant clones were isolated on a bacterial lawn. Gene replacement by targeting vectors was verified by PCR. Introduction of mutations was also verified by cutting PCR fragment with BspEI. The genomic region surrounding the *h2bv3* locus was sequenced to confirm mutations.

To express H2Bv3-Flag in *Dictyostelium* the vector pDBSrH2B-3XFlag, which drives C-terminally tagged H2Bv3 from the constitutive actin 15 promoter on an extrachromosomal vector, was transfected into Ax2 cells and pools of blasticidin resistant colonies analysed. Mutation of E18A, E19A or both were introduced by replacing the coding sequence of the N-terminal region of H2Bv3 following PCR amplification of the sequences including the relevant mutations generated in pQE30 (see below).

### Subcellular fractionation and immunoprecipitation

Chromatin enriched fractions and whole cell extracts were prepared as previously described^43^. Histone enriched acid extracts were prepared as described previously^55^. Briefly, untreated or Phleomycin-treated cells were washed in ice-cold KK2 buffer, and then resuspended in nuclear extraction buffer (50 mM Tris pH8.0, 10 mM NaCl, 3 mM MgCl2, 3 mM CaCl2, 0.5 M sorbitol, 0.6% Triton X 100, Complete protease inhibitor cocktail (Roche), phosphatase inhibitor cocktail 2 and 3 (Sigma), 10 μM Benzamide, and 200 μM DEA) at a cell density of 1 × 10^8^/ml, incubated with rotation at 4 °C for 15 minutes, and the nuclei pelleted by centrifugation at 2300 xg for 5 minutes. Extracted nuclei were resuspended in nuclear extraction buffer containing 4 M urea, and 2% β-mercaptoethanol, agitated at 4 °C for 15 minutes, and pelleted again by centrifuging at 2300 × g for 5 minutes. Isolated nuclei were then resuspended in 0.4 N HCl at a density of 5 × 10^8^/ml, and mixed overnight by rotation at 4 °C. Acid extracted histones were harvested by centrifugation at 16000 xg, and the supernatant precipitated by addition of x6.5 volume of acetone, incubating at −20 °C for 2 hours, and centrifuging at 16000xg for 15 minutes at 4 °C. The pellet was washed twice with ice-cold acetone, dried, and resuspended in 8 M Urea supplemented with 5% beta-mercaptoethanol for further analysis.

For immunoprecipitation, chromatin extracts from cells expressing Flag-tagged H2Bv3 were boiled in 2xSDS sample buffer (100 mM Tris-HCl pH6.8, 4% SDS) to disrupt protein-protein and protein-DNA interactions. Samples were diluted 10 times with IP buffer (50 mM Tris pH8, 200 mM NaCl, 1 mM EDTA, 1 mM DTT, 10% glycerol, 1% triton X-100, phosphatase inhibitor cocktail 2,3 [Roche], 200 μM DEA, 500 μM benzamide, protease inhibitor cocktail tablet [Roche]). Anti-Flag M2 beads (Sigma) were added and the mixture incubated on a rotating wheel for 1 h at 4 °C. Beads were collected by centrifugation band washed 3 times in IP buffer followed by Western blot analysis.

### Sensitivity Assays

Sensitivity assays using germinated spores were performed as described previously^46^. In brief, fruiting bodies were suspended in KK2 containing 0.1% NP-40, and then passed through 19.5-gauge needle to liberate spores. Spores were washed twice by KK2 + 0.1% NP-40, and resuspended in KK2 at the density of 2 × 10^7^ cells/ml. Germination of spores was induced by heat shock at 45 °C for 30 min. Hatched spores were then diluted to 10^6^ cells/ml in a 1:5 ratio of HL5/KK2, and incubated in shaking suspension at 100 rpm for 18 hrs at 22 °C with the concentrations of phleomycin as indicated. 250 cells were plated onto three 140mm Petri dishes with *K. aerogenes* in association with SM agar, and incubated at 22 °C. Colonies on Petri dishes were counted after 3 to 5 days, and cell survival calculated by normalizing with the number of colonies without phleomycin treatment.

### Antibodies

Full length His-tagged H2Bv3 was purified from bacteria under denaturing conditions of 8 M Urea using QIAexpressionist protein purification kit (QIAGEN). Purified protein used to inoculate sheep (Diagnostics Scotland, Edinburgh, Scotland) to produce anti-*Dictyostelium* H2Bv3 anti-serum.

Other antibodies employed in this study are PAR polyclonal antibody (Trevigen; 4336-BPC-100), poly-ADP-ribose binding reagent (Millipore; MABE1031), pan-ADP-ribose binding reagent (Millipore; MABE1016), histone H3 (Abcam; ab1791), γH2AX (Abcam; ab11174), Myc 9E10 (Santa Cruz; sc-40), actin C-11 (Santa Cruz; sc-1615), FLAG M2 (Sigma; F1804), penta-His (Qiagen; 34660), DdKu80^43^.

### Protein expression and purification

H2Bv3 was cloned into pQE30 expression vector to generate N-terminally tagged His6-H2Bv3 and mutation of E18, E19 or both to alanine introduced by site-directed mutagenesis (QuikChange, Agilent Technologies). The primers used to introduce the mutations were ACTCAATCTGGTGCAGAGAAAACCGCTTCA (E18A), ACTCAATCTGGTGAAGCGAAAACCG (E19A) and ACTCAATCTGGTGCAGCGAAAACCG (E18AE19A). Truncation of the first 25 amino acids of H2Bv3 was performed by PCR amplification of the coding sequence without the first 25 amino acids using primer CCAGGATCCATGACCCCAAAAGTAACCAAAACC and reinserting, in frame, into pQE30. All constructs were verified by DNA sequencing.

Expression constructs were introduced into *E. coli* M15 and expression induced with 0.1 mM IPTG for 4 h at 22 °C. Cells were then harvested and resuspended in ice-cold column buffer (50 mM Tris-HCl pH8, 150 mM NaCl, 10% glycerol, 30 mM Imidazole, protease inhibitor cocktail tablet [Roche] and 1 mM PMSF [Sigma]). Cell lysis was performed by sonication. Soluble proteins were then collected following centrifugation of lysates at 15,000 rpm for 30 min and loaded onto a column containing prewashed Ni-NTA beads (Qiagen). After washing with 30 mM, 50 mM and 100 mM Imidazole, H2Bv3 was eluted using column buffer with 200 mM Imidazole, then dialyzed against storage buffer (50 mM Tris-HCl pH8, 10% glycerol), snap frozen in liquid nitrogen and stored at −80 °C.

To generate the catalytic-dead version of Adprt1a (Adprt1a^cd^), H789 and Y823A in the conserved NAD + binding pocket of the catalytic domain were mutated to alanine by the QuickChange method (Agilent Technologies). His-tagged Adprt1a and Adprt1a^cd^ were purified in a similar way to other proteins (see above), although cell lysis was performed using Bugbuster (Merck) to avoid genomic DNA contamination. IMAC buffer (50 mM HEPES pH8, 500 mM NaCl, 10% glycerol, 1 mM DTT, 1 mM Imidazole) was used instead of the column buffer.

### ADP-ribosylation assays

Recombinant His-tagged Adprt1a (100 ng) was mixed with 1.5 mM NAD^+^, 250 μM Biotinylated NAD^+^ (Trevigen) in 50 mM Tris-HCl pH8 and 2 mM MgCl_2_ either with or without 100 μg/ml of sheared salmon sperm DNA. Recombinant His-tagged *Dictyostelium* histone (100 ng) was added where required and the reaction performed at room temperature for 30 mins before termination by addition of 2xSDS loading buffer and boiling for 5 mins. Samples were resolved by SDS-PAGE and transferred to PVDF membrane and the ADP-ribosylation signal detected using HRP-conjugated Streptavidin, followed by ECL. For assays that employed radioactive NAD + where indicated, 100 nM ^32^P-labeled NAD^+^ was used instead of Biotinylated NAD^+^. The ADP-ribosylation of proteins was assessed by separating samples on SDS-PAGE gel, vacuum-drying and exposing to X-ray film.

For de-ADP-ribosylation assays, ADP-ribosylation reactions using Biotinylated NAD^+^ were terminated by adding 10 mM 3-aminobenzamide. Then, 3 μl of 2.4 μM MacroD1 protein or 9 nM PARG enzyme (Trevigen) were added to the reaction either undiluted or diluted 1:5 and 1:10. Mixtures were incubated at 37 °C for 30 mins and the reaction stopped by adding 2xSDS loading buffer and boiling for 5 min. The ADP-ribosylation status was then assessed by Western blot.

## Additional Information

**How to cite this article**: Rakhimova, A. *et al*. Site-specific ADP-ribosylation of histone H2B in response to DNA double strand breaks. *Sci. Rep.* **7**, 43750; doi: 10.1038/srep43750 (2017).

**Publisher's note:** Springer Nature remains neutral with regard to jurisdictional claims in published maps and institutional affiliations.

## Supplementary Material

Supplementary Information
