# Supplementary material for: Site-specific ADP-ribosylation of histone H2B in response to DNA double strand breaks
Source: Sci Rep. 2017 Mar 2;7:43750. doi: 10.1038/srep43750 (PMC5333086; doi:10.1038/srep43750)
Supplement: Supplementary Information [file srep43750-s1.pdf]

Site-specific ADP-ribosylation of histone H2B in response to DNA double strand breaks

Alina Rakhimova , Seiji Ura , Duen-Wei Hsu, Hong-Yu Wang, Catherine J. Pears and Nicholas D. Lakin

Department of Biochemistry, University of Oxford, South Parks Road, Oxford OX1 3QU, UK

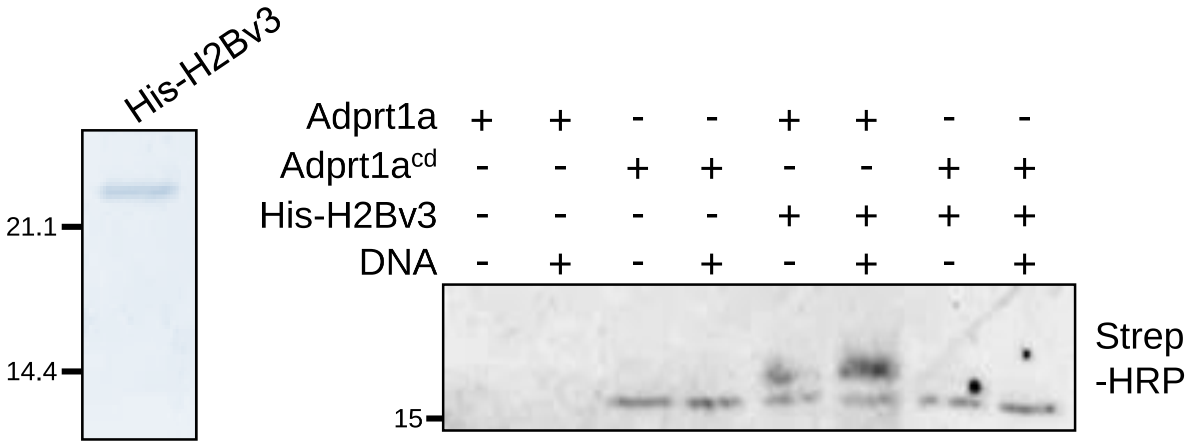

Appendix Figure S1: Catalytic activity of Adprt1a is required for ADP-ribosylation of H2Bv3 *in vitro*
His-tagged H2Bv3 was expressed and purified from bacteria (left panel). ADP-ribosylation assays were performed in the presence or absence of wild-type or catalytic dead (Adprt1a^{cd}) Adprt1a and sheared salmon sperm DNA as indicated. Biotin conjugated NAD+ was employed in reactions to allow the recognition of ADP-ribosylated proteins by western blot analysis using streptavidin-conjugated HRP.

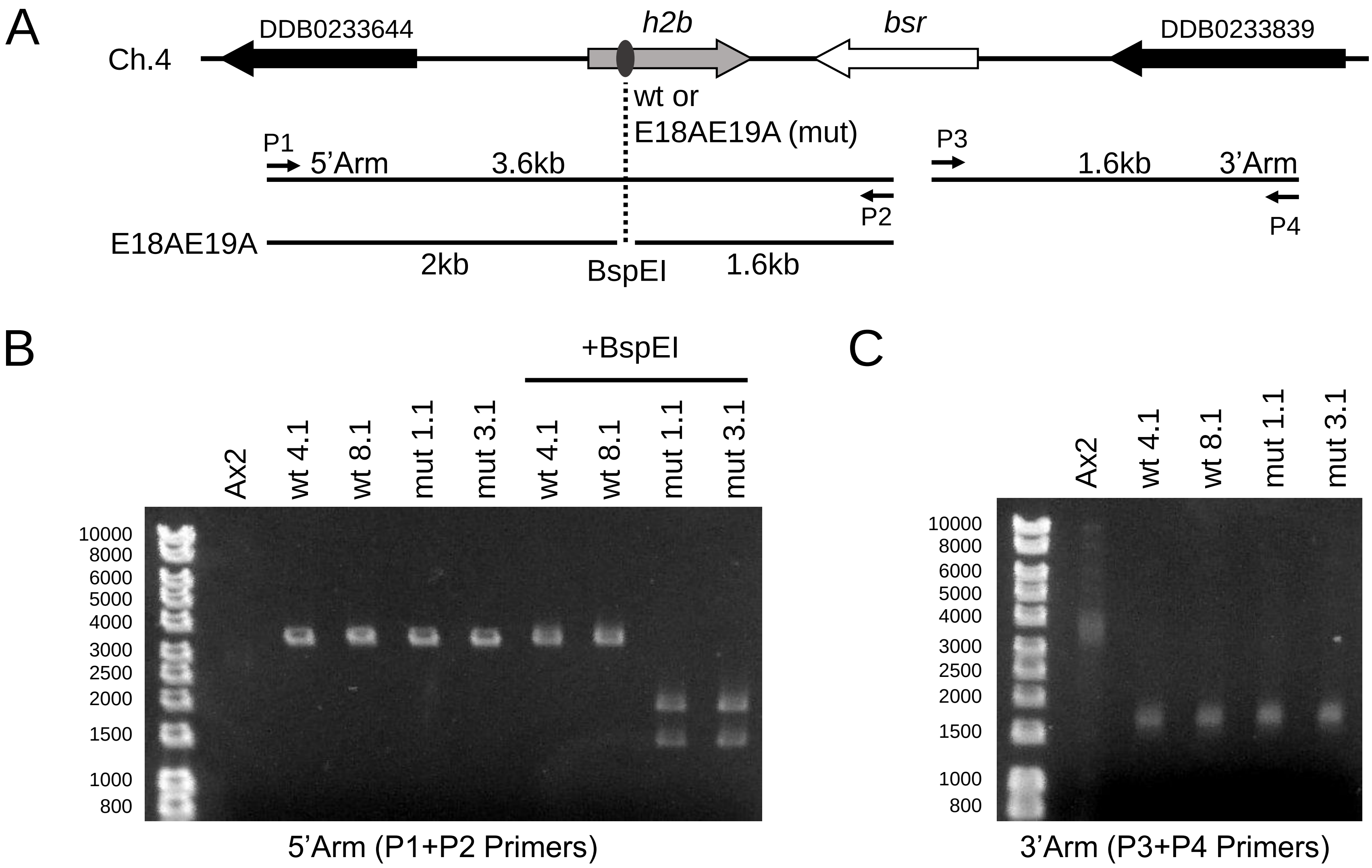

Appendix Figure S2: Verification of *h2bv3*^{E18AE19A} and *h2bv3*^{wt} strains

A. Strategy for PCR-based screening of gene replacement strains. Primers were designed against genomic sequences outside the targeting vector regions (P1 and P4), and sequences inside the *bsr* gene (P2 and P3). E18AE19A mutation also introduces a silent mutation to introduce an additional BspEI site. Expected PCR fragment sizes are indicated. **B. and C.** PCR-amplified fragments were separated by 1% agarose gel electrophoresis. 5'Arm fragments were also cut by BspEI to confirm introduction of E18AE19A mutation.

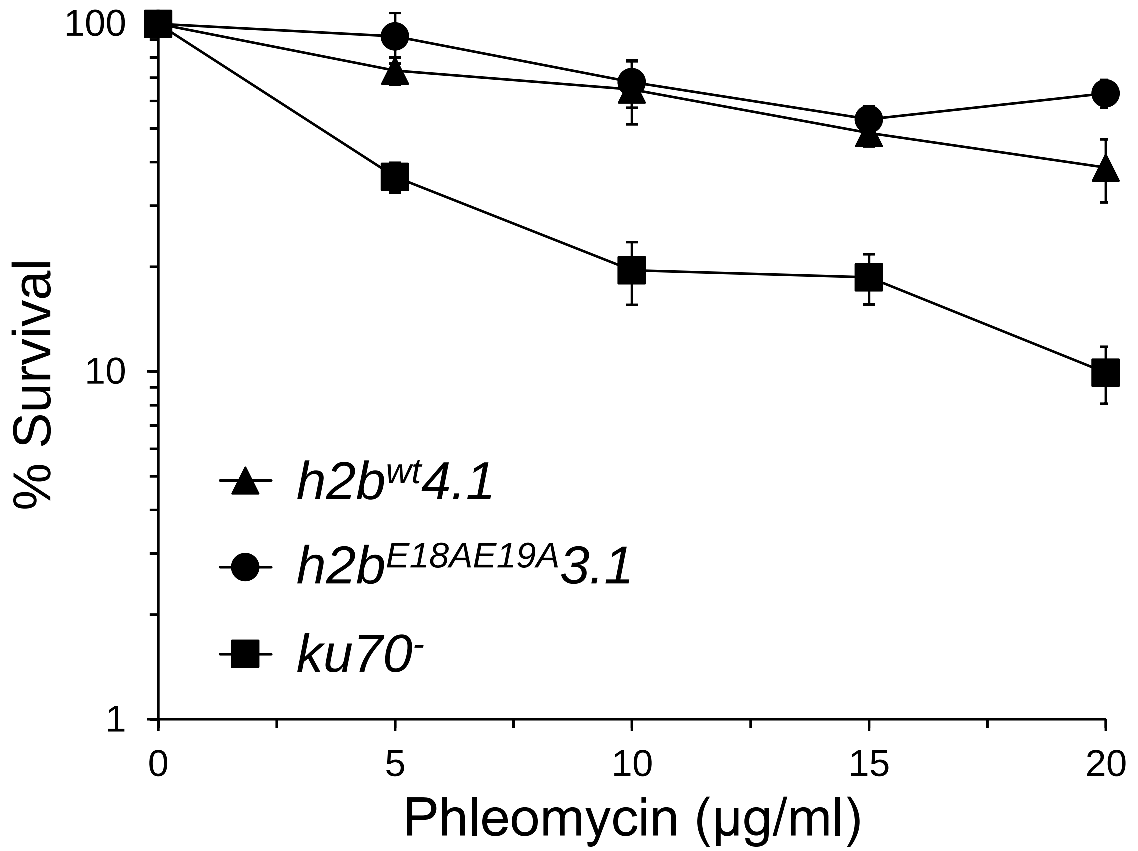

Appendix Figure S3: The *h2bv3^{E18AE19A}* strain is not sensitive to phleomycin relative to *h2bv3^{wt}* control cells during spore germination. Spores from the *h2b^{wt}*4.1, *h2b^{E18AE19A}*3.1 and *ku70^{-}* strains were germinated and then exposed to phleomycin for 18 hrs and cell survival measured as described in Materials and Methods. Error bars represent the SEM from three independent experiments.
